# An in vitro study measuring marginal gaps of inlay restorations fabricated from different CAD-CAM materials after thermocycling

**DOI:** 10.1186/s12903-023-03687-4

**Published:** 2023-12-06

**Authors:** Ahmed Ismail Taha, Mona Elshirbini Hafez

**Affiliations:** 1 Department of Prosthodontic, Faculty of Dentistry, Kafrelsheikh University, Mubark Road, Kafr Abu Tabl, Kafrelsheikh Governorate, Kafrelsheikh, 33511 Egypt; 2 Department of Conservative, Faculty of Dentistry, Kafrelsheikh University, Kafrelsheikh, Egypt

**Keywords:** CAD-CAM, Ceramics, Margin, SEM, Inlay

## Abstract

**Background:**

Many monolithic machined materials have been introduced and provided a suitable mechanical and physical properties for inlay restorations. However, there is shortage in the studies evaluating the marginal adaptation using these materials.

**Purpose:**

This study aimed to compare the effect of fabricating inlay restorations from 3 different CAD-CAM materials on marginal gaps before and after thermocycling.

**Materials and methods:**

Sixty human premolars were randomly divided into 3 groups (n = 20) according to the material used: (e.max CAD, Ivoclar AG, Schaan, Liechtenstein), (HC, Shofu, Koyoto, Japan) and (Brilliant Crios, Coltene, Altstätten, Switzerland) (n = 20). A scanning electron microscope (SEM) (JSM- 6510 lv, JEOL, Tokyo, JAPAN) was used to for measuring the marginal gaps after cementation of inlay restorations. The magnification was adapted to 250x. Marginal gaps were revaluated with SEM after thermocycling. The temperatures of baths were 5 and 55 °C was applied for a total of 5000 cycles. All data were statistically analyzed by using ANCOVA to demonstrate if there were any statistically significant differences between the gap measures after thermocycling of the three independent (unrelated) groups. A Bonferroni adjustmen was used to perform post hoc analysis (α = 0.05).

**Results:**

Post-intervention marginal gap was statistically significantly lower in group EX (110.8 μm) which was statistically significant compared with group SF (112.5 μm) (mean difference=-1.768, *P* = .007) and group BR (113 μm) (mean difference=-2.272, *P* = .001), however, in. comparing SF and BR groups, there was no significant difference (mean difference=-0.5, *P* = .770).

**Conclusions:**

Thermocycling affected the marginal gaps of composite based restoration and resin-modified ceramics widely. However, it had a very small effect on glass ceramics marginal adaptation.

**Clinical implications:**

The marginal gaps of CAD-CAM inlays varied according to material used (ceramic based, combination, or resin based). Thermocycling has a minor effect on the marginal adaptation of lithium disilicate glass-ceramic inlays, where it affected the margin of resin-modified ceramic and composite based inlays greatly. Using lithium disilicate glass-ceramic might improve the clinical longevity of inlay restored teeth.

## Introduction

Tooth-colored restorations are considered a primary requirement for dentist and patient because of biocompatibility and esthetics characteristics which are the main reasons for using dental ceramics as a preferred material for inlays in present time [1].

Ceramic inlay is accepted clinically as a restoration for posterior teeth with extended coronal destruction and became a substitute for dental alloys [2]. Ceramic restorations may fail because of secondary caries, ceramic restoration fracture or supporting tooth structure, loss of marginal integrity or postoperative sensitivity [3] To stabilize the sound tooth substance of teeth with mesio-occluso-distal (MOD) extension preparations with lost mesial and distal contacts and weak remaining tooth structure, inlay ceramic restorations are indicated in such scenarios [4].

The bond between ceramic inlays and the tooth structure along with the mechanical friction are major roles of success for inlays retention [5]. Thus, the type of ceramic should be considered for an effective bonding. Both, lithium disilicate ceramic and feldspathic have shown an excellent bond with tooth structure that is suitable for inlay restorations [6]. Moreover, survival of inlay restorations depends on the marginal accuracy which is considered a principal factor of success. A defective marginal integrity increases the plaque accumulation, promote periodontal problems [7], causes microleakage [8], secondary caries at restoration margins and finally pulpal involvement [9]. However, properly fit margins for inlay restorations is difficult to achieve, because of considerable inherent properties of adhesives, such as relatively high polymerization shrinkage, low degradation resistance and high coefficient of thermal expansion [7].

In addition to conventional impressions, digital impressions are used as they are faster with reduction of the workflow steps and better tolerability by the patient. Moreover, the virtual scan file can be transferred to the lab through certain applications, without fabrication of a die or providing clinical bite registration [10].

According to the type of restoration, the highest clinically marginal gap width values that is acceptable was suggested in the previous literature [11, 12]. The maximum marginal gap for CAD-CAM restorations ranging between 75 and 160 μm were showed in studies as clinically acceptable marginal integrity [13, 14]. Although, a wide range of marginal gap values was described in research was due to restoration type, margin location [15, 16], the accuracy of fit in CAD-CAM systems may be affected by scanning procedures, software program used for restoration designing, milling machine and shrinkage compensation [17]. The inlay preparation design is more complex than crown preparation, which affect the accuracy of scanning which in turn can disrupt the adaptation of inlay restorations in some areas [18, 19].

Ceramic materials are known to be brittle and more liable for fracture, still harder than composite based materials, therefore ceramics are more wear resistant. On the contrary, they may cause wear of the opposing tooth surface [20]. Resin-modified ceramic materials deliver the characteristics of both materials (ceramics and composite); they have an elastic modulus close to that of the tooth structure. Moreover, they can be easily adjusted, repaired, or modified in the same way as composite materials [21].

Therefore, this study aimed to analyze the effect of varieties of CAD-CAM materials on marginal gap of inlay restorations before and after thermocycling. The null hypothesis supposed firstly that “there would be no significant difference in the marginal gap between the milled CAD-CAM inlays from 3 CAD-CAM” and secondly that “This would not change after thermocycling”.

## Materials and methods

This in-vitro research was submitted to the ethical committee of the Faculty of oral and dental medicine and surgery, Kafrelsheikh University, Kafrelsheikh, Egypt and approved with number (MKSU/22-11-1). The sample size was determined based on the given formula used for studies with purposive sampling. Assuming 80% power, 5% significance, and a 95% confidence interval, the required sample size for each group was nine. A total of 20 samples were included in each group. Sixty recently extracted human premolars were extracted at local oral and maxillofacial surgery clinics under approval of local institutional review board–protocol due to periodontal and orthodontic indications. An informed consent for using extracted teeth was signed and obtained from patients. The inclusion criteria of selected teeth were intact maxillary premolars fully developed with crown size of 7 ± 0.5 mm width. The teeth were examined with magnifying loupes to ensure that it is free from fracture lines, cracks, caries, non-carious defects, and restorations. Selected teeth were stored in a 0.9% NaCl containing 0.1% thymol at room temperature until use [22].

Class II MOD cavity was prepared with rounded internal angle and without bevel. Cavity preparations were done by a single operator (AT) with the suggested series of specific diamond burs 6º taper (Inlay Preparations Set 4261, Komet, Lemgo, Germany) accompanied with continuous water-cooling. A surveyor (Paraskop, Bego, Bremen, Germany) was used to standardize the cavity preparations dimensions; it has a milling arm that was designed as a multifunction arm for the facility of preparations in different directions, and a holder at the end of arm for a handpiece used for preparation. A motor was built in the base to control the handpiece. The principles of ceramic and indirect composite mesio-occluso-distal (MOD) inlay preparation mentioned in literature [23] were followed in cavity preparation. The depth of pulpal floor was 2.5 mm starting from the occlusal surface, the occlusal isthmus width was 2.5 mm, and the diameters of the mesial and distal boxes buccolingually were the same as the occlusal isthmus width. In each box, the gingival floor depth was 1.5 mm mesiodistally, and 2 mm height for the axial wall. The cavosurface angles of all margins were prepared with 90°.

After preparation, specimens were divided randomly into 3 groups according to CAD/CAM materials were used (n = 20): group EX (e.max CAD, Ivoclar AG, Schaan, Liechtenstein), Group SF (HC, Shofu, Koyoto, Japan) and Group BR (Brilliant Crios, Coltene, Altstätten, Switzerland) as shown in Table 1.

**Table 1:** CAD-CAM materials used in the present study

Group	Block used	Manufacturer	Composition	
EX	e.max	Ivoclar AG	Lithium disilicate glass ceramic	70 vol% lithium disilicate and glass ceramic
SF	Shofu block HC (SH)	Shofu	Resin-modified ceramic	61% zirconium silicate, UDMA, TEGDMA, Micro fumed silica.
BR	Brilliant Crios	Coltene	Reinforced composite	29.3 wt% Cross-linked methacrylates and 70.7 wt%Amorphous silica

An intraoral scanner (Medit i700, MEDIT Corp, Seoul, Republic of Korea) was used to scan all prepared specimens. Standard Tessellation Language (STL) files were transferred for designing the virtual inlay restorations by software program (DentalCAD 3.0 Galway 2021, exocad, Darmstadt, Germany). The spacer thickness was virtually set to be 50 μm of the internal and marginal discrepancy. The designing of all restorations was according to the related occlusal anatomy of the tooth being restored. STL files of inlay restoration were transferred to a 5-axis milling machine (Coritec 250i, imes-icore GmbH, Eiterfeld, Germany) to be milled from the following CAD-CAM materials: IPS e.max CAD (block: LT A2/C14), HC Shofu (disk: A2 Diameter 98 mm, Thickness 14 mm), and Brilliant Crios (disk: A1 HT diameter 98.5 mm, height 14 mm). In group EX, (IPS e.max CAD) endocrowns were subjected to the process of crystallization with furnace (Vita Vacumat 6000 M; VITA Zahnfabrik GmbH, Bad Säckingen, Germany) following the manufacturer instructions, while in both groups SH (HC Shofu) and BR (Brilliant Crios), endocrowns did not require any crystallization firing. After milling, inlay restorations were evaluated on the prepared teeth, and pressure areas were identified by using a water-soluble pressure indicating paint (PIP; Keystone Industries, Singen, Germany). A finishing green diamond point (DCB, Schleifer, Komet Dental, Lemgo, Germany) was used to remove all detected pressure areas until complete seating was verified by using a sharp explorer at different marginal sites. Improvement of marginal gaps was noticed by 2 clinicians. Before the endocrown cementation, a cotton moistened with alcohol was used to clean all specimens.

The protocol of cementation was different according to the material being cemented and the manufacturer instructions. For group EX, the inlay restoration was surface treated with hydrofluoric acid gel 4.5% (Porcelain etch, Ultradent Products, Cologne, Germany) for 20 s, washed, dried, and silane coupling agent was applied. For group SF, the intaglio surface was sandblasted with 50 μm aluminum oxide (Al2O3) particles, cleaned with phosphoric acid etchant gel 37% and finally treated with HC primer and air dried. For group BR, the intaglio surface was sandblasted using 50 μm aluminum oxide (Al2O3), surface was cleaned and dried. For the prepared tooth surface, the enamel was selectively etched with 37.5% phosphoric acid (Ultra-Etch, Ultradent Products, Cologne, Germany) for 30 s, rinsed, and dried.

A self-adhesive resin luting agent (Rely-X Unicem, 3 M ESPE, St. Paul, USA) was used for cementation. Inlay restorations was cemented perpendicular to pretreated prepared tooth surface using finger pressure to stabilize and allow the restoration to be fully seated. Excess cement was removed with sharp explorer. A visible light cure with low power 650 mW/cm^2^ (Bluephase G2, Ivoclar AG Schaan, Liechtenstein) was applied for 20 s on each surface. The cement margin was finished using flexible polishing discs (Sof-LexXT Pop-On, 3 M ESPE AG, Seefeld, Germany).

**Fig. 1:**
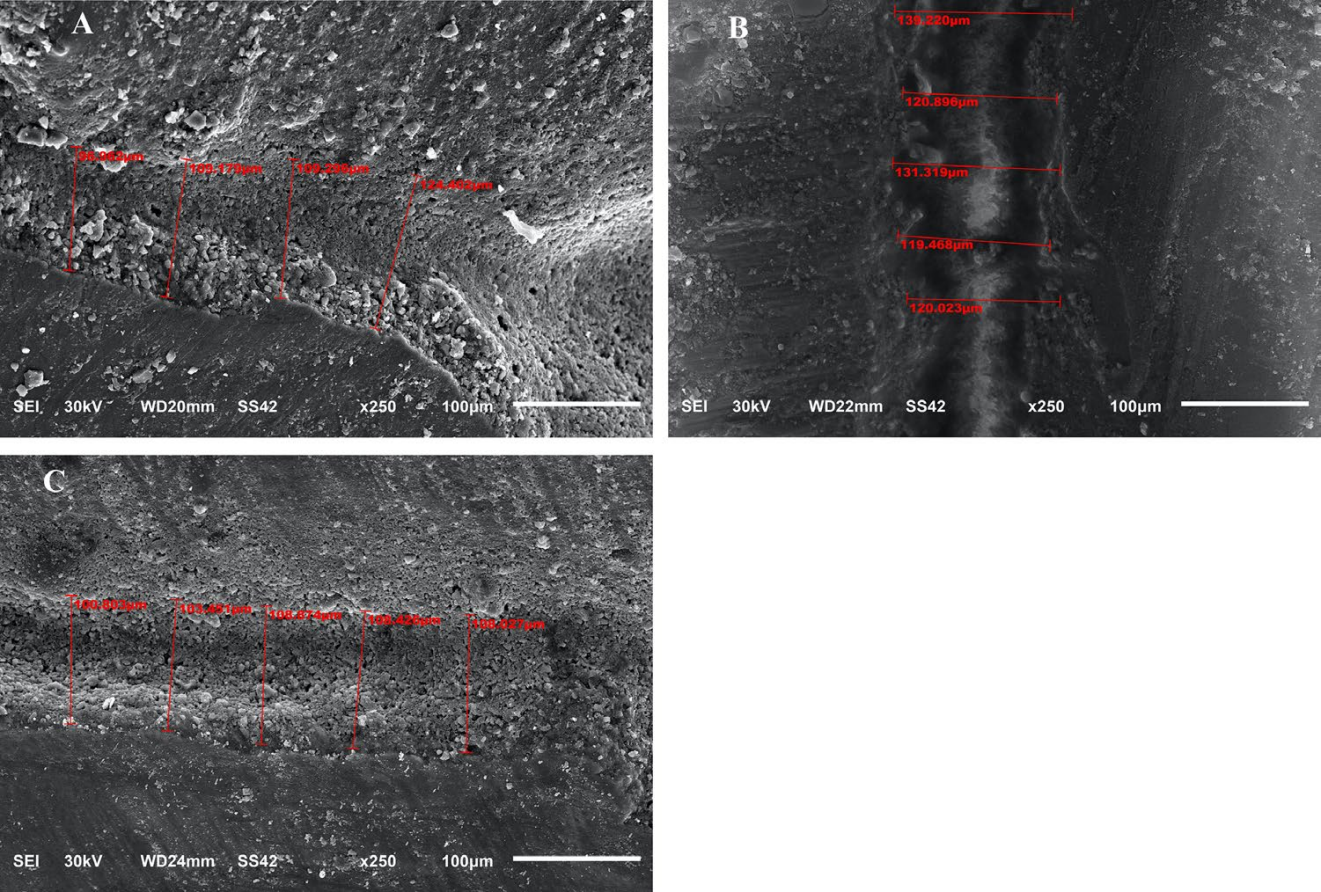
** A**–**C**, SEM measurements at magnification x250 of marginal gaps for CAD-CAM, computer aided design and computer-aided manufacture endocrown restorations in 3 groups (EX, SF, and BR) (**A**, **B**, and **C** are showing measurements for 3 groups before thermocycling)

**Fig. 2:**
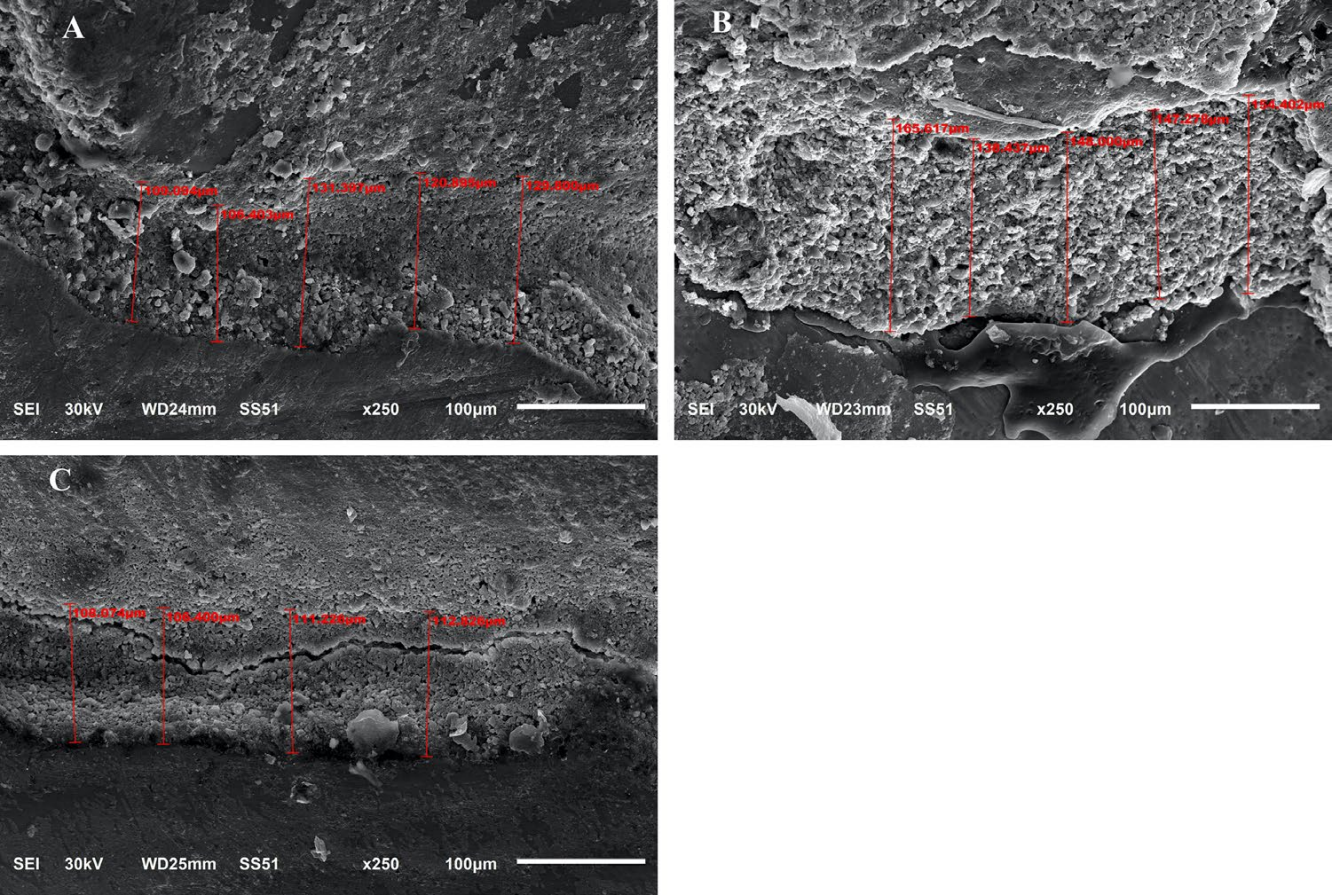
** A**–**C**, SEM measurements at magnification x250 of marginal gaps for CAD-CAM, computer aided design and computer-aided manufacture endocrown restorations in 3 groups (EX, SF, and BR) (**A**, **B**, and **C** are showing measurements for 3 groups after thermocycling)

After cementation, the marginal gaps were measured by a scanning electron microscope (SEM) (JSM- 6510 lv; JEOL, Tokyo, JAPAN) in Mansoura Microscopy Center, Faculty of Agriculture, Mansoura University. Samples were coated with 24 kt gold by a metallizer (SPI-MODULE, sputter coater, SPI supplies, West Chester, USA). Firstly, the image with whole coronal portion of tooth with restoration was automatically appear on computer monitor attached to the microscope. The magnification was adapted to 250x. After that, selected areas at (gingival and axial walls) were determined and the marginal gaps were measured in micrometers as shown in (Fig. 1A–C). The average of gingival and axial marginal gaps was calculated by using 30 measures for each wall. The samples were exposed to thermocycling with baths temperatures 5 and 55 °C, for a total of 5000 cycles. The bath time for each temperature was 20 s, and the time of transfer between baths was 2 s. After thermocycling, the marginal gaps were revaluated with SEM in the same way mentioned before as shown in (Fig. 2A–C).

### Statistical analysis

The collected data were statistically analyzed with a software program (IBM SPSS Statistics, v20.0, IBM Corp, New York, USA) (α = 0.05). Initially, the normality was tested for the quantitative data with Shapiro-Wilk test showing that data being normally distributed if *P* > .05. Presence of significant outliers (extreme values) was tested for by inspecting boxplots. Quantitative data were demonstrated as mean ± standard deviation (normally distributed). One-way ANOVA was used to compare between quantitative data of 3 groups. An ANCOVA was used to demonstrate if there were any statistically significant differences between the adjusted marginal gap means of the three independent (unrelated) groups. A Bonferroni adjustmen was used to perform post hoc analysis. Through all used tests, results were considered as statistically significant if *P* value ≤ 0.05.

**Fig. 3:**
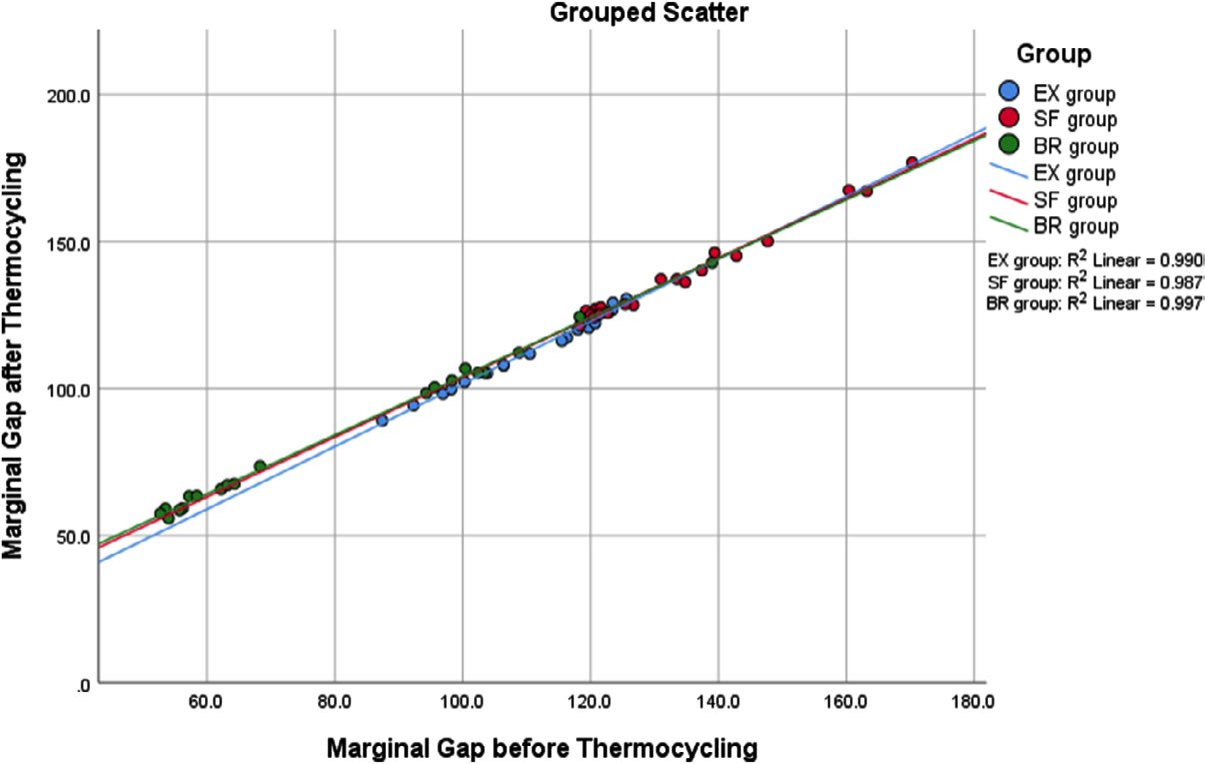
Grouped Scatterplot for marginal gap

**Fig. 4:**
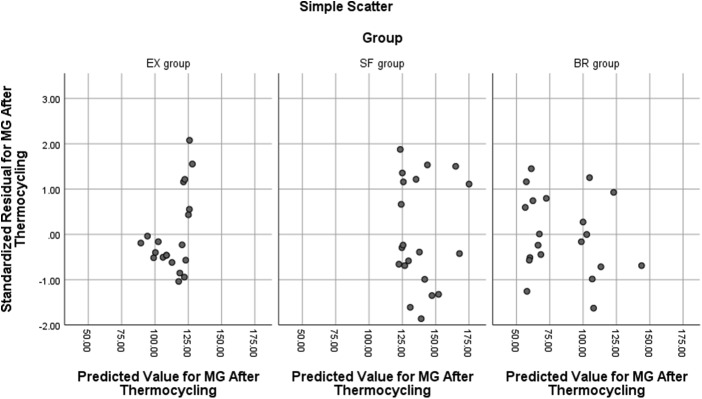
Simple scatterplot predicted values versus standardized residuals

One-way ANCOVA reveled the following assumptions: Linearity assumptionas shown in Fig. 3, all groups have a linear relationship between the covariate and the dependent variable. Homogeneity of regression slopes showed homogeneity of regression slopes as the interaction term was not statistically significant, F (2, 54) = 1.770, *P* = .180. Normality showed that, the standardized residuals for the interventions were normally distributed, as assessed by Shapiro-Wilk test (*P* > .05) except for EX group (*P* = .013). However, one-way ANCOVA is robust to deviations from normality. Standardized residuals for the overall model were not normally distributed, as assessed by Shapiro-Wilk test (*P* = .022). There was homoscedasticity, as assessed by visual inspection of the standardized residuals plotted against the predicted values as shown in Fig. 4. There was homogeneity of variances, as assessed by Levene test of homogeneity of variance (*P* = .060). Finally, there were no outliers in the data, as assessed by no cases with standardized residuals greater than ± 3 standard deviations.

## Results

Results of the present study revealed that smallest mean marginal gaps values were noted in BR group (80.3 μm) followed by EX group (111.3 μm) then SF group (133.9 μm), before thermocycling.

Table 2 showed that, the effect of groups EX, SF, and BR interventions on post-intervention marginal gap after controlling for pre-intervention marginal gap. After adjustment for pre-intervention marginal gap, there was a statistically significant difference in post-intervention marginal gap between the interventions, F (2, 56) = 10.884, *P* < .001, partial η^2^ = 0.280.

**Table 2:** Adjusted and unadjusted intervention means and variability for post-intervention marginal gap with pre-intervention marginal gap as a covariate

Group	Unadjusted		Adjusted		F	p-value	Partial η^2^
	Mean	SD	Mean	SE			
EX	113.6	12.4	110.8	0.353	10.884	< 0.001	0.280
SF	138.2	16.2	112.5	0.449			
BR	84.5	26.5	113.0	0.469			

*Note*: SD = standard deviation. SE = standard error. Partial η^2^ is a measure of effect size. The test of significance is one-way ANCOVA

Post-intervention marginal gap was statistically significantly low in group EX (110.8 μm) compared with group SF (112.5 μm) (mean difference=-1.768, *P* = .007) and group BR (113 μm) (mean difference=-2.272, *P* = .001), however, there was no significant difference between SF and BR groups (mean difference=-0.5, *P* = .770). It is also noticed that crack occurred in cement layer of groups BR and SF after thermocycling.

## Discussion

This in vitro study investigated the behavior of 3 types of CAD-CAM materials for fabricating inlay restorations regarding the marginal integrity before and after thermocycling. The first null hypothesis was rejected as there was significant difference between marginal gaps in different inlays groups. The second null hypothesis was rejected since the marginal gaps changed in all groups after thermocycling.

To possibly simulate the clinical procedures, natural teeth with similar dimensions were selected and the preparation was standardized by using a dental surveyor. Digital impression technique started to replace conventional techniques that allow direct scanning of prepared teeth and eliminate clinical errors. For this purpose, CAD-CAM scanner was used in the present study for precise procedure [24].

Direct evaluation through a SEM was the selected method for marginal fitting evaluation, which allowed obtaining standardized measurements by positioning the restored teeth in a base [13, 25]. The drawback of such technique is that the differentiation between the tooth structure and the most inferior part of the finish line margin was difficult. On the other hand, it has many advantages such as being a fast technique and its low cost; as the technique does not require additional procedures such as sectioning of specimens. In addition, the risk of cumulative errors is lower than other techniques with multi-step procedures [26, 27] Moreover single operator, who made all measurements and was blinded to the restoration materials.

Additionally, the measurements of marginal gaps were decided to be done after cementation of inlay restorations to simulate the clinical conditions [13, 28] A cement space of 50 μm was used for creating an even layer of cement [14], and this cement space was approved by previous studies to have a marginal fit values within clinical acceptable limit [13, 29] Resin-based composite cements are usually the selected adhesive cement used for inlay restorations cementation. Since satisfying marginal conditions achieved after cementation is one of the most related factors for long-term clinical success of fixed restorations.

Marginal gaps are affected by multiple factors such as the design of preparation [30], fabrication technique [31], gaps measurement technique [14], and the materials used [32]. Different monolithic CAD-CAM materials became viable for construction of inaly restoration [33]. Three different CAD-CAM materials were selected for this study (e.max CAD, HC Shofu, and Brilliant Crios) which are based on different structures (lithium disilicate glass ceramic, resin-modified ceramic, and resin nanoceramics). Each of which has different properties and behavior inside the oral cavity.

Machinability of blocks differs from one material to another according to the ease of milling of each material, and that depends on the brittleness index, which is affected by the fracture toughness and hardness, microstructure of the material, and chipping factor [34]. Low hardness and modulus of elasticity were found to be accompanied by greater amounts of material being removed during milling [35]. Additionally, the other factor is the flexure resistance which affects the resistance to crack propagation during milling [36]. The materials also are affected by temperature changes in the oral cavity and in this study, thermocycling was done 5000 cycles with temperature of baths 5 and 55 °C and dwell time 20s to simulate the temperature changes of an oral cavity and 6 months of clinical service [37, 38].

Results of the present study revealed that smallest mean marginal gaps values were for group BR (80.3 μm) followed by group EX (111.3 μm) and group SF (133.9 μm), before thermocycling. All marginal gaps were within acceptable clinical marginal gaps as demonstrated in previous studies between 75 and 160 μm [13, 14]. This finding coincides with the results of studies by Francesco et al. [39], and Sağlam et al. [40] who evaluated marginal gaps of CAD-CAM materials (lithium disilicate versus composite and zirconia). This difference may be due to better machinability of resin materials which results from lower hardness value and modulus of elasticity, and that results in more accurate marginal adaptation of the resin matrix material when compared to ceramic. In addition to the process of crystallization can potentially affect the marginal fit. As investigated by Gold et al. [41] who assessed the marginal fit of CAD-CAM-fabricated lithium disilicate crowns both before and after the crystallization process. They found a significant difference in marginal discrepancy, between measurements before (42.9 mm) and after crystallization (57.2 mm). Where it was not reproduced for group SF. Although it has polymer matrix, but it had the highest marginal gap values. This may be due to to the fact that differences in the resin matrix size, type, and composition of the particles used as charge, these dispersed particles on the milled surface are easily cleared by sandblasting which results in differences in gaps measurements [42].

However, marginal gaps of all groups after thermocycling had been changed. Where the change in marginal gaps was statistically significantly lower in group EX compared to group SF (mean difference=-1.768, *P* = .007) and group BR (mean difference=-2.272, *P* = .001) but not between groups SF and BR (mean difference=-0.5, *P* = .770). This may be due to resin matrix composition which is affect by thermocycling more than glass ceramic, and that resulted in dimensional changes and marginal gaps increased in both inlays groups (SF and BR) more than the increase of marginal gap in EX group. This dimensional change caused the crack in cement layer that has been noticed in results. This in agreement with the results of Diana Lopez et al. [43] who conclude that the marginal gaps of two CAD-CAM glass ceramic increased after thermocycling. In contrary, Kun Qian et al. [44] demonstrated that 10,000 cycles had no effect on marginal gap of different hybrid ceramic materials.

There were some limitations in this study; firstly, it was an in vitro study, which is different from a clinical study situation, where the scanning of prepared teeth would be affected by saliva, and limited accessability for the scanner inside the oral cavity which make scanning less precise. Secondly, the study didn’t evaluate the internal adaptation of restorations. Lastly, the potential accuracy of milling machine may not be the best.

## Conclusions

Based on the findings of this in vitro study, the following conclusions were drawn:

1. Marginal gaps vary depending on different materials used. BR showed the lowest marginal gaps followed by EX, and the largest marginal gaps was noted in SF.
2. All marginal gaps of different groups were within the acceptable range.
3. Thermocycling affected the marginal gaps of composite based restoration and resin-modified ceramics widely. However, it had a very small effect on glass ceramics marginal gap.

## Data Availability

The datasets generated and/or analysed during the current study are not publicly available due to [the research is not published yet] but are available from the corresponding author on reasonable request.
